# NF-κB/RelA-PKM2 mediates inhibition of glycolysis by fenofibrate in glioblastoma cells

**DOI:** 10.18632/oncotarget.4444

**Published:** 2015-06-30

**Authors:** Dongfeng Han, Wenjin Wei, Xincheng Chen, Yaxuan Zhang, Yingyi Wang, Junxia Zhang, Xiefeng Wang, Tianfu Yu, Qi Hu, Ning Liu, Yongping You

**Affiliations:** ^1^ Department of Neurosurgery, The First Affiliated Hospital of Nanjing Medical University, Nanjing 210029, China

**Keywords:** fenofibrate, PPARα, RelA, PKM2, Warburg effect

## Abstract

Aerobic glycolysis (production of lactate from glucose in the presence of oxygen) is a hallmark of cancer. Fenofibrate is a lipid-lowering drug and an agonist of the peroxisome proliferator-activated receptor alpha (PPARα). We found that FF inhibited glycolysis in a PPARα-dependent manner in glioblastoma cells. Fenofibrate inhibited the transcriptional activity of NF-κB/RelA and also disrupted its association with hypoxia inducible factor1 alpha (HIF1α), which is required for the binding of NF-κB/RelA to the *PKM* promoter and PKM2 expression. High ratios of PKM2/PKM1 promote glycolysis and inhibit oxidative phosphorylation, thus favoring aerobic glycolysis. Fenofibrate decreased the PKM2/PKM1 ratio and caused mitochondrial damage. Given that fenofibrate is a widely used non-toxic drug, we suggest its use in patients with glioblastoma multiforme (GBM).

## INTRODUCTION

The metabolism of tumor cells is characterized by elevated glucose uptake and lactate production even in the presence of oxygen, a phenomenon known as “Warburg effect” or “aerobic glycolysis” [1, 2].

As the rate-limiting final step of glycolysis, Pyruvate kinase isoenzyme (PK) catalyzes the conversion of phosphoenolpyruvate and adenosine diphosphate (ADP) to pyruvate and adenosine triphosphate (ATP) [3]. Pyruvate kinase isoenzyme type M2 (PKM1) and Pyruvate kinase isoenzyme type M2 (PKM2) are two isoforms of PK. PKM1 and PKM2 exclusively contain exon 9 and exon 10, respectively, and are both encoded by the *PKM* gene using the same promoter [4]. The single exon difference endows the enzymes with distinct expression patterns and functions. PKM2 is expressed in embryonic, proliferating, and cancer cells, whereas PKM1 is expressed in normal differentiated tissues. High ratios of PKM2/PKM1 promote glycolysis but inhibit oxidative phosphorylation [5, 6]. To ensure a high ratio of PKM2/PKM1 in cancer cells, three heterogeneous nuclear ribonucleoproteins (hnRNPs) proteins, including polypyrimidine tract-binding protein (PTB, also known as hnRNPI), hnRNPA1, and hnRNPA2, alternatively splice transcripts of the *PKM* gene and facilitate the generation of PKM2 [7]. Furthermore, NF-κB/RelA upregulates *PKM* transcription, depending on its interaction with HIF1α [8]. Expression of PKM2 is critical for cancer cell growth, including glioblastoma [9]. Switching from PKM2 to PKM1 in human lung cancer cells reduced the ability to form tumors in nude mouse xenografts [10].

The nuclear factor kappa enhancer-binding protein (NF-κB) family is composed of structurally homologous transcription factors, including NF-κB1 (p50 and its precursor, p105), NF-κB2 (p52 and its precursor, p100), RelA (p65), RelB, and c-Rel [11]. All five family members have a Rel homology domain that is responsible for DNA binding and dimerization between different or identical family members, leading to homomeric or heteromeric binding of the subunits. In an inactive state, NF-κB is sequestered in the cytoplasm as homomeric or heteromeric and IκB subunits. In response to stimulation, NF-κB, predominantly the p65/p50 dimer, is released from binding to IκB and transfers to the nucleus and then binds to a specific consensus sequence in the DNA, which results in gene transcription [12]. Activation of NF-κB is associated poor survival [13].

The peroxisome proliferator-activated receptor α (PPARα) is a nuclear receptor, which belongs to the superfamily of steroid hormone receptors [14]. PPARα has anti-inflammatory properties due to inhibition of NF-κB/RelA [15]. Fenofibrate (FF) is a ligand of PPARα, has been shown to exert interesting anticancer properties [16, 17], which is rapidly converted to fenofibric acid (FA) *in vivo* by tissue and plasma esterases before entering the cell [18]. Wilk et al. reported that FF induced metabolic catastrophe and glioblastoma cell death involving the AMPK-mTOR-autophagy pathway [19].

Here we show that FF not only inhibits glucose uptake and lactate production but also induces mitochondrial damage in human glioblastoma cells. FF also causes NF-κB/RelA-dependent downregulation of PKM2 expression, depending on PPARα.

## REULTS

### FF suppresses glioblastoma cells glycolysis

**Figure 1:**
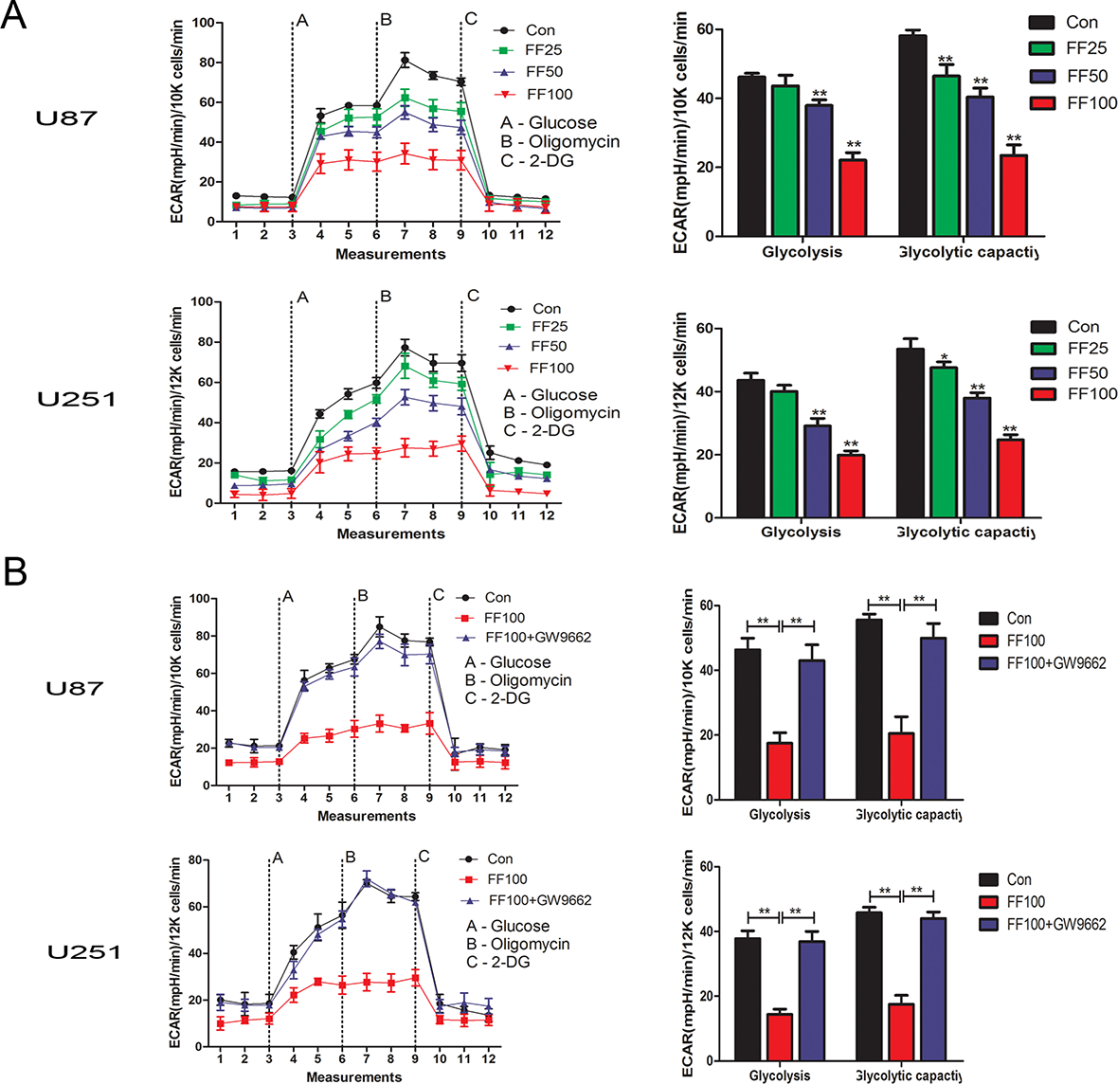
Inhibition of cell glucose metabolism by FF in the human glioblastoma cells **A.** Cells were treated with various concentrations (25, 50 and 100 μM) of FF. After 48 h, ECAR was measured by the Glycolysis Stress tests. **B.** GW9662 (10 μM), a PPAR inhibitor, restores FF-induced the inhibition of cell glucose metabolism. *P < 0.05, **P < 0.01. Results are representative of at least three independent experiments.

We determined the effect of FF on glycolysis of U87 and U251 glioblastoma cells using the XF analyzer to perform glycolysis stress test assays. Both glioblastoma cell lines were treated FF at different concentrations including 0 μM (Con), 25 μM (FF25), 50 μM (FF50), and 100 μM (FF100) for 48 h. The results shown in Figure 1A indicate that FF significantly inhibited glycolysis in a dose-dependent manner (25–100 μM). Next, we also explored whether PPARα is involved in its anti-glycolysis action. GW9662, a synthetic PPARγ inhibitor, also blocks PPARα at higher concentrations (10 μM) [20]. As shown in Supplementary Figure S1, FF increased PPARα transcriptional activity in a dose-dependent manner and GW9662 significantly decreased PPARα transcriptional activity. Then ECAR in the presence of FF (FF100) and GW9662 (10 μM) was tested in U87 and U251 glioblastoma cells. The results indicated that the action of FF on glioblastoma cell glycolysis is PPARα dependent (Figure 1B).

### NF-κB/RelA transcriptionally induces PKM2

**Figure 2:**
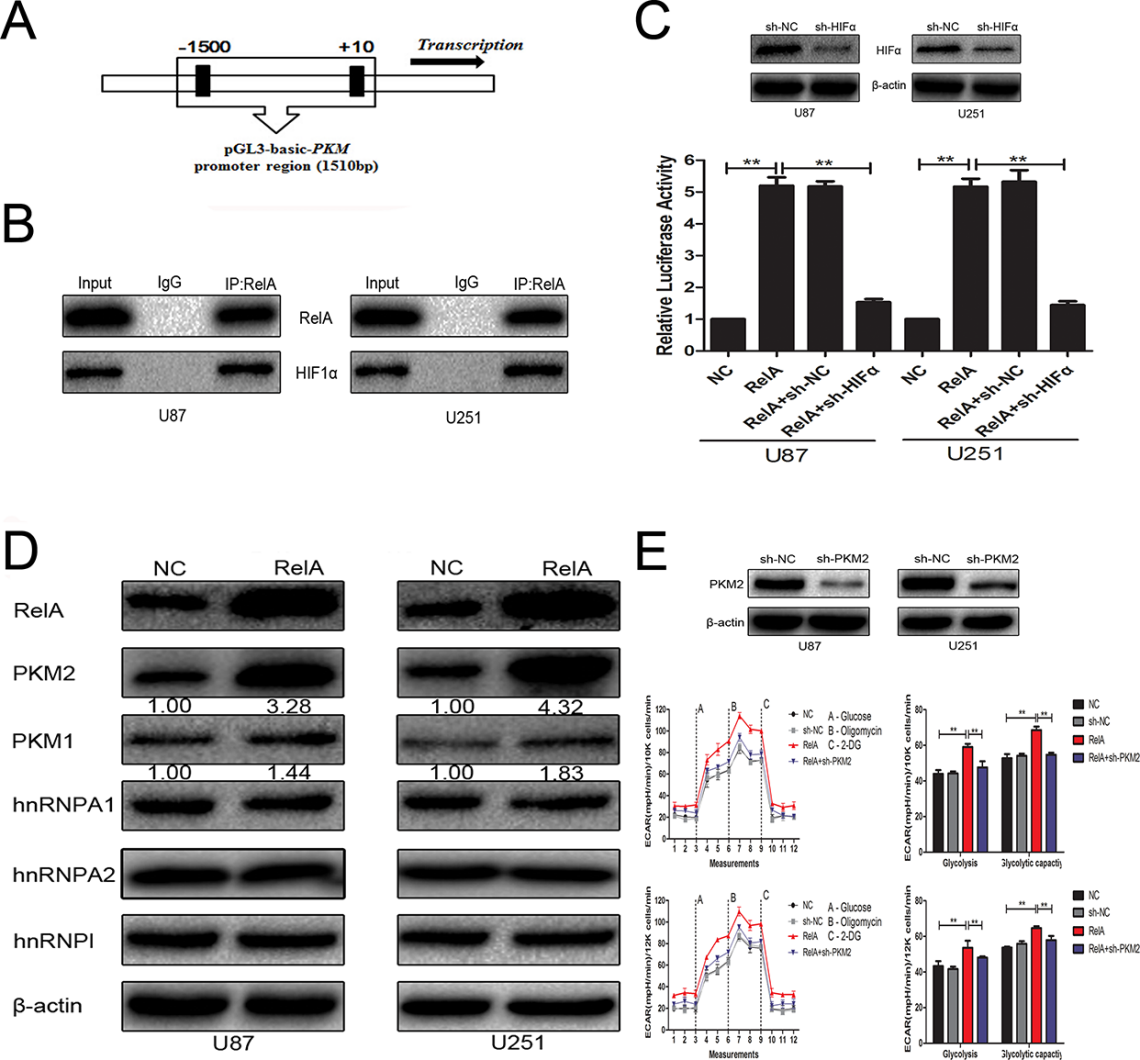
PKM2 was positively regulated by NF-κB/RelA at the transcriptional level **A.** Schematic of putative NF-κB/RelA-binding motifs in the *PKM* promoter sequence. **B.** Co-IP demonstrates that NF-κB/RelA physically interacts with HIF1α in the nucleus. **C.** *PKM* promoter-containing pGL3-basic plasmid, RelA overexpression plasmid, and sh-HIF1α were co-transfected into cells. Luciferase assays confirmed the enhancement of *PKM* promoter activity after RelA overexpression in the presence of HIF1α. **D.** Western blots identified hnRNPA1, hnRNPA2, hnRNPAΙ, PKM1, and PKM2 expression changes following transfection with RelA overexpression plasmid. β-actin is shown as a loading control. **E.** ECAR was measured in glioma cell lines after transfection with RelA overexpression plasmid alone or in combination with sh-PKM2. **P < 0.01. Results are representative of at least three independent experiments.

The *PKM* promoter, containing NF-κB/RelA-binding sites, was cloned into the luciferase reporter plasmid pGL3 (Figure 2A). The plasmid was co-transfected with the RelA overexpression plasmid alone or in combination with sh-HIF1α in U87 and U251 glioblastoma cells. Results in Figure 2B and Figure 2C demonstrate that the overexpression of NF-κB/RelA enhanced the activity of luciferase construction in the presence of HIF1α, which was consistent with the findings of a previous study [8]. The HnRNP protein family could alternatively splice transcripts of the *PKM* gene. We checked the effect of NF-κB/RelA on the expression of PKM1, PKM2, hnRNPΙ, hnRNPA1, and hnRNPA2 in U87 and U251 glioblastoma cells. Western blot analysis depicted in Figure 2D revealed that the expression level of PKM2 was remarkably increased, whereas that of PKM1 was slightly increased. On the other hand, the HnRNPs protein level was not significantly affected by RelA overexpression. Furthermore, NF-κB/RelA obviously promoted glioblastoma cell glycolysis depending on PKM2 (Figure 2E) but not PKM1 (data not shown).

### NF-κB/RelA is involved in FF-induced inhibition of glycolysis

**Figure 3:**
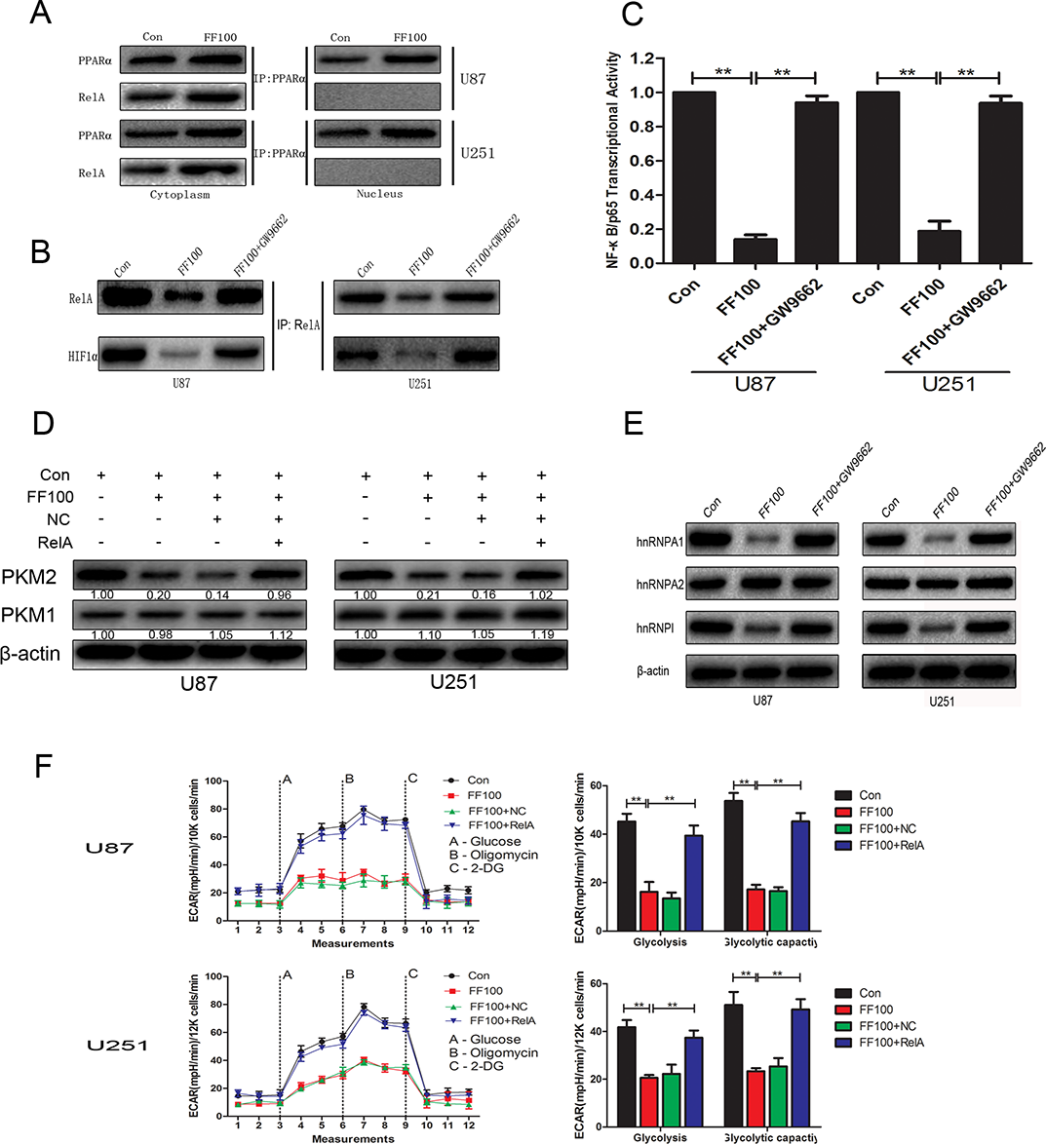
NF-κB/RelA is involved in FF-induced inhibition of glycolysis **A.** and **B.** Cells were treated with FF (100 μM), followed by Co-IP. FF promoted PPARα/RelA complex formation in the cytoplasm. Furthermore, FF not only inhibited nuclear translocation of NF-κB/RelA but also induced dissociation of NF-κB/RelA and HIF1α in the nucleus. **C.** FF inhibited NF-κB/RelA transcriptional activity in a PPARα-dependent manner. **D.** and **E.** Western blots identified hnRNPA1, hnRNPA2, hnRNPAΙ, PKM1, and PKM2 expression changes following treatment with FF (100 μM) alone or in combination with RelA overexpression plasmid. β-actin as a control should be included. **F.** RelA overexpression obviously reversed FF-induced inhibition of glycolysis. **P < 0.01. Results are representative of at least three independent experiments.

It has been reported that PPARα interacts with RelA and the interaction region contains RHD, which mediates DNA binding [15]. In glioblastoma cells, the interaction of PPARα and RelA only appeared in the cytoplasm. Treatment with FF (FF100) significantly promoted the interaction of PPARα and RelA in the cytoplasm (Figure 3A). FF-mediated activation of PPARα not only notably suppressed NF-κB/RelA transcriptional activity but also promoted the separation of NF-κB/RelA and HIF1α (Figure 3B and Figure 3C). Following this, to determine whether NF-κB/RelA is responsible for FF-mediated anti-glycolysis in glioblastoma cells, we performed glycolysis stress test assays. The results in Figure 3F demonstrated that NF-κB/RelA overexpression obviously reversed FF-induced inhibition of glycolysis. Interestingly, the PKM2 protein level was remarkably attenuated, whereas the expression of PKM1 did not change (Figure 3D). FF-induced suppression of hnRNPA1 and hnRNPΙ may contribute to this interesting result (Figure 3E).

### FF promotes mitochondrial damage

**Figure 4:**
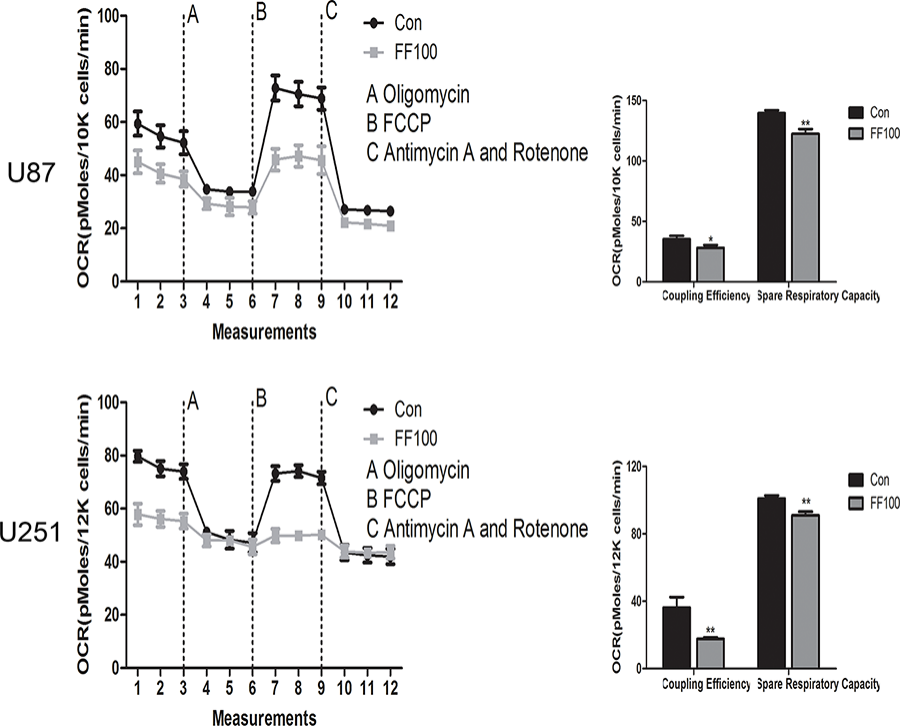
OCR was measured by the cell mito stress tests in glioma cell lines treated with FF (100 μM) *P < 0.05, **P < 0.01. Results are representative of at least three independent experiments.

**Figure 5:**
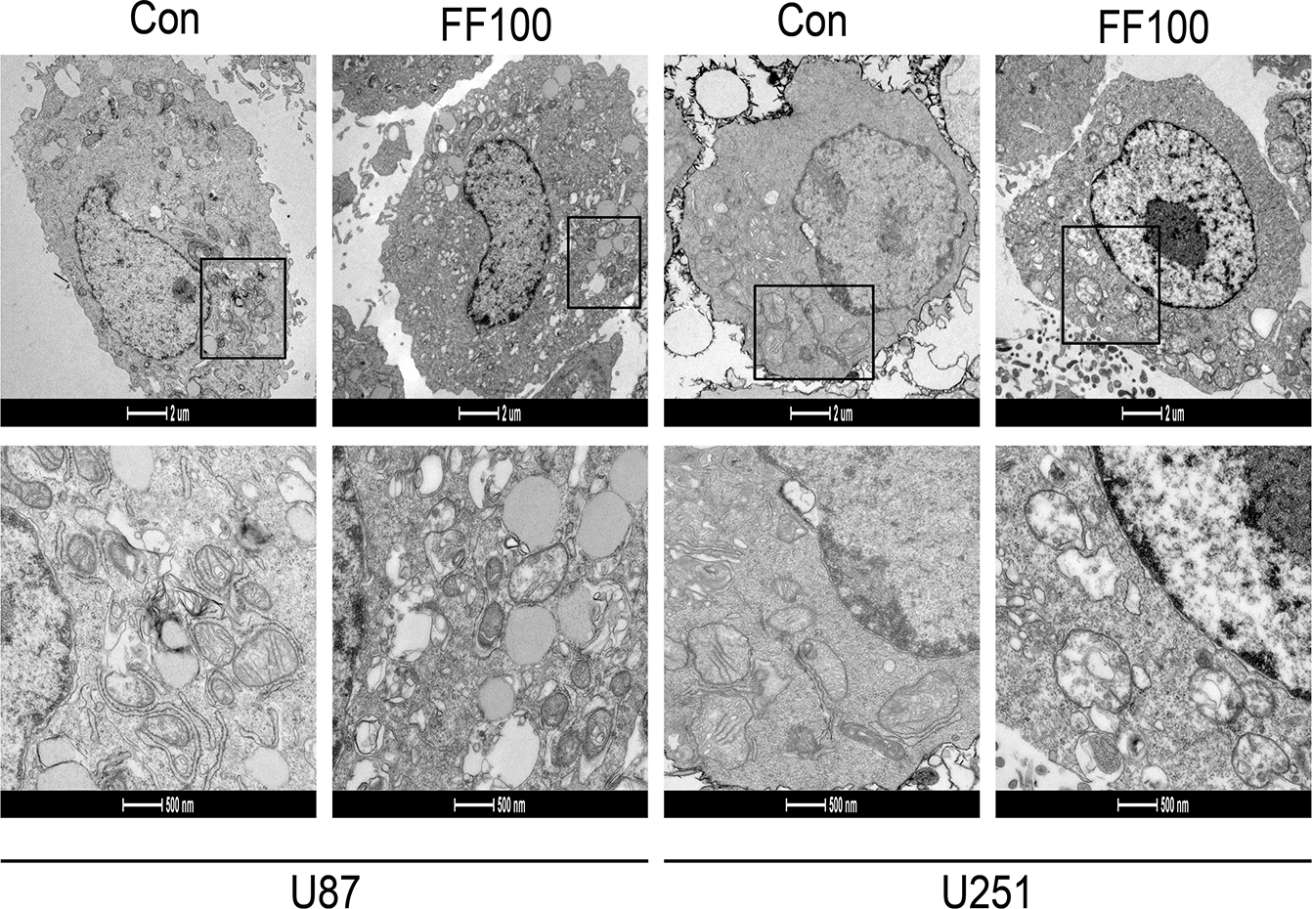
TEM images showing mitochondrial structural damage induced by FF (100 μM) in human glioblastoma cells

High ratios of PKM1/PKM2 elevate the glucose flux into oxidative phosphorylation and then enhance O_2_ consumption. Although FF significantly reversed ratios of PKM2/PKM1 in the glioblastoma cells, O2 consumption was incredibly reduced (Figure 4). The ultrastructural morphology of mitochondria in glioblastoma cell was observed by a routine TEM (Figure 5). TEM images showed that FF promoted mitochondrial structural damage, including mitochondrial swelling and disruptions or integration of mitochondrial crest, compared with the control group, which may lead to their dysfunction.

### FF suppresses glioma growth of glioma *in vivo*

**Figure 6:**
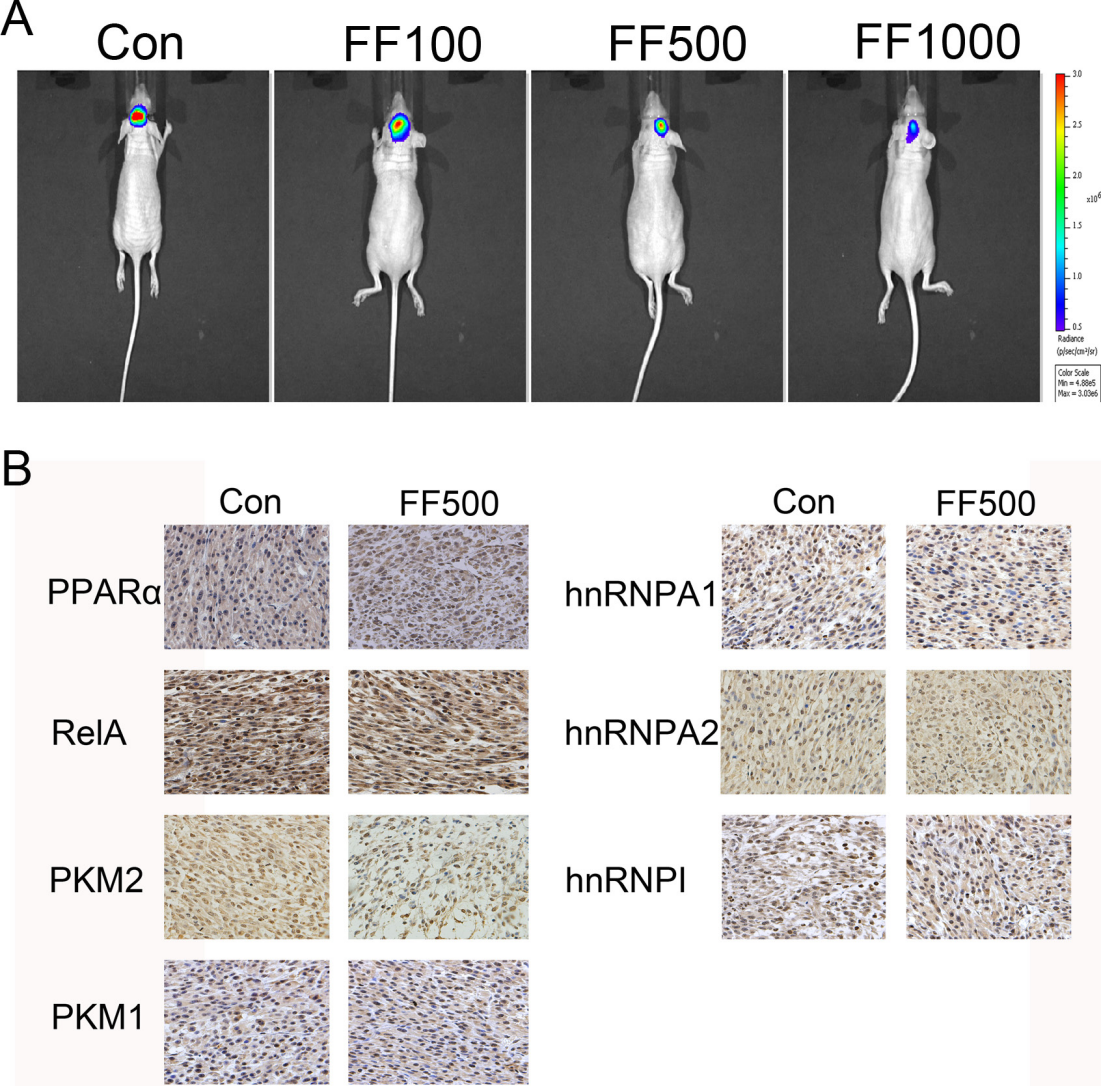
Intracranially-delivered FF inhibits glioblastoma tumor growth **A.** Representative images of intracranial tumor growth after intracranial injection of FF. **B.** The expression of PPARα, RelA, PKM2, PKM1, hnRNPA1, hnRNPA2 and hnRNPAΙ was examined by IHC staining of sections from a glioma xenograft model in nude mice.

**Figure 7:**
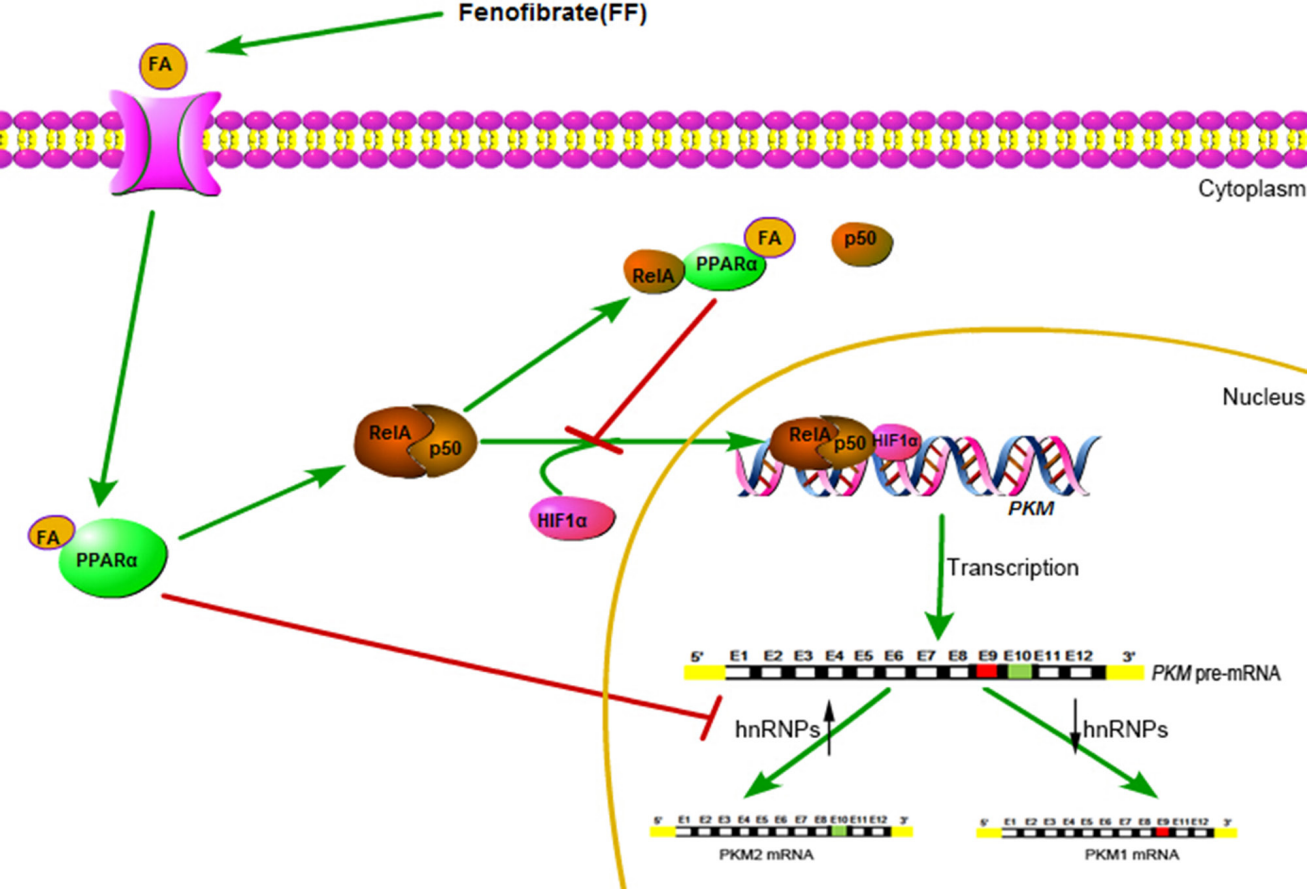
A schematic diagram for FF-induced inhibition of glucose metabolism

Considering the remarkable anti-glioblastoma effects of FF *in vitro*, we extended our investigation to test if FF could attenuate glioblastoma growth *in vivo* using nude mice. Bioluminescence imaging showed inhibition of the tumor growth in FF-treated group compared with the control group (Figure 6A). We also evaluated PPARα, RelA, PKM1/2 and hnRNPs protein expression by Immunohistochemical (IHC) staining in a nude mouse glioma xenograft model to study the correlation among them. The results in Figure 6B revealed that the nuclear expression level of PPARα was increased and that of RelA was decreased. PKM2, hnRNPA1 and hnRNPΙ expression was significantly decreased, whereas PKM1 and hnRNPA2 had no obvious change. These results *in vivo* and vitro provide demonstration of the role of FF in the regulation of the PPARα/RelA/PKM2 pathway in glioma (Figure 7).

## DISCUSSION

Proliferating cancer cells require not only ATP but also large amounts of glucose carbons for macromolecular synthesis [21]. Therefore, cancer cells metabolize glucose by glycolysis instead of oxidative phosphorylation even when oxygen is abundant, which has been termed aerobic glycolysis [1].

Fenofibrate (FF) is used to treat hyperlipidemia and hypercholesterolemia [22]. FF has antiproliferative, antimetastatic, and pro-apoptotic effects in tumors of neuroectodermal origin, including melanoma, medulloblastoma, and glioblastoma [23–25]. In our study, we confirmed that FF inhibits glioblastoma glycolysis in a dose-related manner depending on PPARα activation. The role of NF-κB activation during inflammatory response has been intensively studied [26]. PPARα represses inflammatory genes that can be ascribed to the PPARα antagonistic action against NF-κB/RelA [15]. Furthermore, NF-κB/RelA is implicated in many hallmarks of cancer, including proliferation, prevention of apoptosis, and promotion of angiogenesis and metastasis [27, 28]. NF-κB/RelA promotes aerobic glycolysis through transcriptional activation of Glut3 [29] and PKM2 [8]. In the present study, FF inhibited the transcriptional activity of NF-κB/RelA and dissociated RelA and HIF1α, which is required for the binding of NF-κB/RelA to the *PKM* promoter and PKM2 expression. In addition, NF-κB/RelA-dependent *PKM* transcription acts in coordination with splicing of pre-mRNA, which is mediated by FF-induced downregulation of hnRNPA1 and hnRNPΙ, leading to reduced expression of PKM2 but not PKM1. Thus, NF-κB/RelA-PKM2 is involved in FF-induced inhibition of glycolysis.

The high ratio of PKM1/PKM2 favors oxidative phosphorylation and enhances O_2_ consumption [5]. Mitochondria produce ATP through oxidative phosphorylation [30]. FF increased the ratio of PKM1/PKM2, unimaginably accompanied with the reduction of O_2_ consumption. Previous studies have shown that the agents including FF hampered mitochondrial respiration [31, 32]. In our study, FF damaged mitochondria. Scatena et al. suggested possible molecular mechanisms (ROS generation) of mitochondrial impairment [33].

GBM is characterized by rapid growth, resistance to radio- and chemotherapy and relentless invasion of the central nervous system, and it remains practically incurable [34]. NF-κB/RelA is a potential target for therapeutic intervention in GBM [35]. Here we reveal a mechanism of inhibition of glycolysis and NF-κB/RelA-PKM2 by FF in GBM cells. These findings suggest potential application of FF in GBM.

## MATERIALS AND METHODS

### Cell culture and transfection

The human U87 and U251 glioblastoma cell lines were purchased from the Chinese Academy of Sciences Cell Bank and grown in Dulbecco's modified Eagle's medium (DMEM) supplemented with 10% fetal bovine serum (FBS). The PKM2-short hairpin RNA (sh-PKM2) and HIF1α-short hairpin RNA (sh-HIF1α) oligonucleotide were designed and cloned into vector U6/GPF/Neo by GenePharma. Blank vector U6/GPF/Neo was used as a negative control (sh-NC). The recombinant plasmid for pcDNA3.1 vector, which contains the ORF of human RelA (RelA), was purchased from Genechem (Shanghai, China). The blank vector pcDNA3.1 was used as a negative control (NC). All plasmids were transfected into cells using Lipofectamine 2000 Transfection Reagent (Invitrogen, USA) according to the manufacturer's instructions.

### Glycolysis stress test

The extracellular acidification rate (ECAR) was measured using the Seahorse XF96 Analyzer Glycolysis (Seahorse Bioscience, USA), which calculates the net production and extrusion of protons into the extracellular medium. Treated and untreated cells were seeded in XF 96-well plates and incubated overnight at 37°C under 5% CO2 humidified atmosphere. Initially, the cells were incubated in the glycolysis stress test medium without glucose, and ECAR was assessed. Following this, D-glucose (10 mM), oligomycin (1 μM), and 2-deoxyglucose (100 mM) were injected in turn, and ECARs was assessed. The key parameters of glycolytic function, glycolysis and glycolytic capacity, were calculated by the XF Glycolysis Stress Test software.

### Cell mito stress test

The oxygen consumption rate (OCR), an indicator of mitochondrial respiration, was measured using the Seahorse XF96 Analyzer (Seahorse Bioscience, USA). Treated and untreated cells were seeded in XF 96-well plates and incubated overnight at 37°C under 5% CO2 humidified atmosphere. Initially, the cells were incubated in the normal medium, and OCR was assessed. Following this, oligomycin (2.0 μM), carbonyl cyanide-p-trifluoromethoxyphenylhydrazone (FCCP) (0.3 μM), and antimycin A and rotenone (0.5 μM) were injected in turn, and OCRs were assessed. The key parameters of mitochondrial function, coupling efficiency and spare respiratory capacity, were calculated by calculated by the XF Cell Mito Stress Test software.

### Electron microscope

Treated and untreated cells were fixed in 2.5% glutaraldehyde and then dehydrated and embedded. The ultrathin slices of approximately 50–100 nm thickness were observed under a transmission electron microscope (TEM) (JEOL, Japan) after double staining with uranyl acetate and lead citrate.

### Luciferase reporter assay

A 1.5 kb fragment of human *PKM* promoter region containing p65-binding sites was constructed into pGL3-basic plasmids, as described previously (Invitrogen, USA) [8]. The cells were co-transfected with luciferase reporter plasmids, sh-HIF1α, or RelA overexpression plasmids. Cells were collected after 24 h transfection and luciferase activity was measured using the Dual-Luciferase Reporter Assay System (Promega, USA).

### PPARα transcription factor assay

PPARα transcription factor assay was performed as previously described [36].

### NF-κB/RelA transcription factor assay

After FF treatment, nuclear proteins were extracted using the Nuclear/Cytoplasmic Fractionation Kit (KenGEN, China). Equal amounts of protein were used to determine NF-κB/RelA transcriptional activity depending on the NF-κB/RelA Transcription Factor Assay (Abcam, USA) according to the manufacturer's instruction. The absorbance was determined using a microplate reader (Bio-Rad, USA) at 450-nm wavelength.

### Co-immunoprecipitation (Co-IP)

Co-IP is an effective means of quantifying protein–protein interactions in cells. First, subcellular fractionation was applied to separate cytoplasmic and nuclear proteins using the Nuclear/Cytoplasmic Fractionation Kit (KenGEN, China). Following this, equal amounts of nuclear/cytoplasmic proteins were labeled using anti-PPARα (Abcam, USA) or anti-RelA (CST, USA) following overnight incubation at 4°C. The protein–antibody immunoprecipitates were collected by protein A/G plus-agarose (Santa Cruz, USA). Following the final wash, the samples with 1 × SDS were boiled. The PPARα and RelA proteins were analyzed by Western blotting.

### Western blot analysis

All proteins were extracted after lysing cells in RIPA lysis buffer (KenGEN, China). Equal amounts of protein were separated by SDS-PAGE, followed by electrotransfer onto polyvinylidene difluoride membranes (Thermo Scientific, USA). Membranes were blocked for 2 h with 5% nonfat milk and then incubated at room temperature with primary antibody, followed by incubation with a secondary antibody for 2 h. Immunoblot analysis used the following primary antibodies: PPARα (1:1000; Abcam, USA); hnRNPA1 (1:1000; Abcam, USA); hnRNPA2 (1:1000; Abcam, USA); hnRNPΙ (1:5000; Abcam, USA); RelA (1:1000; CST, USA); HIF1α (1: 200; Santa Cruz, USA); PKM2 (1:1000; CST, USA); PKM1 (1:1000; SIGMA, USA); and β-actin (1:1000; CST, USA).

### Xenograft tumor assay

All described procedures involving experimental animals were performed in accordance to standard guidelines under a protocol approved by Nanjing Medical University. Female nude mice at 4–6 weeks of age were used in this experiment. To establish intracranial gliomas, 0.5 × 10^5^ U87 cells stably expressing luciferase reporter (U87-Luc) were implanted stereotactically. Following the tumor cell implantation, mice were injected intracranially with 5 μl of FF (100, 500 and 1000 μM) in DMSO. Mice were imaged for Fluc activity using bioluminescence imaging on 15 days initial cell implantation. Prior to imaging, each mouse received an intraperitoneal injection of D-luciferin (10 μl/g).

### Immunohistochemistry staining

Paraffin-embedded xenograft tumor sections were incubated with diluted primary antibodies against PPARα, RelA, hnRNPA1, hnRNPA2, hnRNPΙ, PKM2 and PKM1 overnight at 4°C. Subsequently, the sections were incubated with a biotinylated secondary antibody (1:200 dilution, Gene Tech, China) at room temperature for 1 h and then the sections were incubated with ABC-peroxidase for 1 h, washed with PBS and stained with diaminobenzidine for 5 min, counterstained with hematoxylin (Gene Tech, China). Six randomly selected visual fields per section were examined by light microscope to evaluate the expression of PPARα, RelA, hnRNPA1, hnRNPA2, hnRNPΙ, PKM2 and PKM1. Sections with no labeling or with fewer than 5% labeled cells were scored as 0. Sections with 5%–30% of cells labeled were scored as 1, with 31%–70% of cells labeled as 2, and with labeling of ≥71% as 3. The staining intensity was scored similarly, with 0 used for negative staining, 1 for weakly positive, 2 for moderately positive, and 3 for strongly positive. The scores for the percentage of positive tumor cells and for the staining intensity were added to generate an immunoreactive score for each specimen. The product of the quantity and intensity scores were calculated such that a final score of 0–1 indicated negative expression (−), 2–3 indicated weak expression (+), 4–5 indicated moderate expression (++), and 6 indicated strong expression (+++). Each sample was examined separately and scored by 2 pathologists. Cases with discrepancies in the scores were discussed to reach a consensus.

### Statistical analysis

Results are provided as means ± standard deviations (SDs) of at least three independent experiments. Statistical comparisons were made using one-way analysis of variance (ANOVA) and Student's *t*-tests (two-tailed) with an SPSS 13.0 software package. *P*-values of < 0.5 were considered significant.

## SUPPLEMENTARY FIGURE


