# Supplementary material for: NF-κB/RelA-PKM2 mediates inhibition of glycolysis by fenofibrate in glioblastoma cells
Source: Oncotarget. 2015 Jun 30;6(28):26119–28. doi: 10.18632/oncotarget.4444 (PMC4694890; doi:10.18632/oncotarget.4444)
Supplement: Supplementary file 1 [file oncotarget-06-26119-s001.pdf]

SUPPLEMENTARY FIGURE

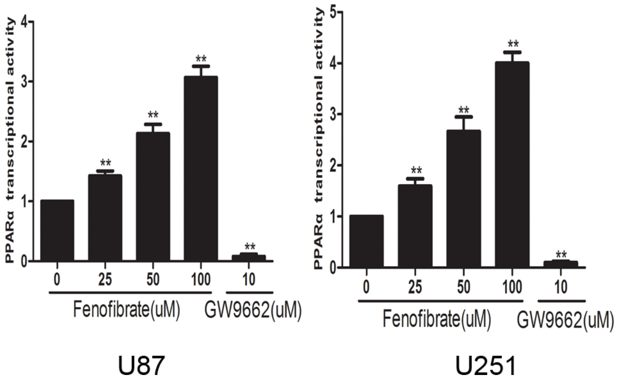

Supplementary Figure S1: FF increases PPARα in a dose-dependent manner and GW9662 (10 µM) decreases transcriptional activity of PPARα.
